# Realization of magnetostructural coupling by modifying structural transitions in MnNiSi-CoNiGe system with a wide Curie-temperature window

**DOI:** 10.1038/srep23386

**Published:** 2016-03-16

**Authors:** Jun Liu, Yuanyuan Gong, Guizhou Xu, Guo Peng, Ishfaq Ahmad Shah, Najam ul Hassan, Feng Xu

**Affiliations:** 1 Jiangsu Key Laboratory of Advanced Micro&Nano Materials and Technology, School of Materials Science and Engineering, Nanjing University of Science and Technology, Nanjing 210094, China; 2 Herbert Gleiter Institute of Nanoscience, Nanjing University of Science and Technology, Nanjing 210094, China

## Abstract

The magnetostructural coupling between structural and magnetic transitions leads to magneto-multifunctionalities of phase-transition alloys. Due to the increasing demands of multifunctional applications, to search for the new materials with tunable magnetostructural transformations in a large operating temperature range is important. In this work, we demonstrate that by chemically alloying MnNiSi with CoNiGe, the structural transformation temperature of MnNiSi (1200 K) is remarkably decreased by almost 1000 K. A tunable magnetostructural transformation between the paramagnetic hexagonal and ferromagnetic orthorhombic phase over a wide temperature window from 425 to 125 K is realized in (MnNiSi)_1−x_(CoNiGe)_x_ system. The magnetic-field-induced magnetostructural transformation is accompanied by the high-performance magnetocaloric effect, proving that MnNiSi-CoNiGe system is a promising candidate for magnetic cooling refrigerant.

Magnetostructural transformation (MST), a coupling between the structural and magnetic transition, attracts considerable attention due to various associated interesting magnetoresponsive effects, such as magnetic shape memory effect^1^, magnetic field-induced strain^2^,^3^, magnetoresistance^4^,^5^ and magnetocaloric effect^6^,^7^,^8^,^9^. These effects show potential applications in sensors^10^, magneto-mechanical devices^11^,^12^, energy-harvesting devices^13^, magnetic cooling refrigeration^14^,^15^, and so on. In order to realize MST in a phase-transition material, a large magnetization difference (ΔM) between the two structural phases is essential and is always pursued to increase the magnetic field-driving capacity^1^. If MST in a given system is tuned to occur between a paramagnetic (PM)/antiferromagnetic (AFM) state and a ferromagnetic (FM) state^1^,^16^, rather than between two FM states^17^, a large ΔM will be gained. As an example, MST is widely observed in Heusler-type magnetic shape memory alloys, which show ferromagnetic martensitic transitions^18^,^19^.

Based on this viewpoint, another type of magnetic shape memory material, the hexagonal MnM′X compound (M′ = Co or Ni, X = Si or Ge) is designed to obtain MST^20^,^21^,^22^,^23^. In stoichiometric MnM′X compounds, the structural transformation takes place from a martensitic-like hexagonal Ni_2_In to an orthorhombic TiNiSi structure during the cooling process^24^. The main challenge in MnM′X compounds is that the structural transformation temperature (T_t_) is usually much higher than the magnetic-ordering temperatures of both hexagonal and orthorhombic phases^25^,^26^,^27^, and the transformation occurs between two PM states^25^,^26^,^27^, resulting in a low ΔM and low magnetic field-driving capacity^17^.

It is known that, in MnM′X compounds, the stability of Ni_2_In and TiNiSi structures is highly dependent on the covalent bonds between M′ and X atoms^28^,^29^, and that between the neighbouring Mn and Mn atoms^16^. The stoichiometric tuning^20^,^23^, foreign-atoms-substituting^16^,^21^,^22^,^30^,^31^ and applied stress^32^ may alter the strength of covalent bonds, thus influence T_t_. For example, by introducing interstitial B atoms or substituting Cu for Mn atoms, T_t_ of MnCoGe can be reduced from 420 K to below Curie temperature of the orthorhombic phase (T_C_) and MST from PM hexagonal to FM orthorhombic phase is realized during cooling^22^,^31^. In the case of MnNiGe system, the stoichiometric tuning can reduce T_t_ from 470 K to below than 200 K and obtain MST from FM hexagonal to AFM orthorhombic phase with decreasing temperature^20^. Moreover, it is reported that if T_t_ lies in the Curie-temperature window (CTW), which is the range between Curie temperatures of the hexagonal and orthorhombic phases^16^,^33^,^34^, the structural transition will couple with magnetic state changes, bringing a large ΔM. The CTW is expected to be broad enough, so that MST and the coupled magnetoresponsive effects can be freely tailored in a large temperature range. However, in MnM′Ge-based compounds, the relatively low magnetic ordering temperatures restrict the further enlargement of CTW.

Aimed at the potential applications with magnetoresponsive effects, a good candidate of MnM′X compound with MST should have both a large ΔM and a wide CTW. As a member of MnM′X family, MnNiSi has a high T_C_ of 622 K^35^, indicating a potential large CTW. However, T_t_ in stoichiometric MnNiSi alloy is as high as 1200 K^24^, which is almost two times higher than those in MnCoGe and MnNiGe^24^,^25^,^26^,^27^. Compared to the previously mentioned alloy systems, to effectively tune down T_t_ to below T_C_ is a big obstacle in MnNiSi system before the realization of MST^36^,^37^. Recently, the isostructural alloying opens up a new feasibility to realize the magnetostructural coupling in MnNiSi-based compounds^16^,^33^,^34^,^38^,^39^,^40^,^41^. By applying this method, the wide CTW can be further obtained. In this work, by Co and Ge co-substitution, we perform the isostructural alloying of MnNiSi and CoNiGe. In this (MnNiSi)_1−x_(CoNiGe)_x_ system, T_t_ is successfully tuned down to below than T_C_, and the tunable MST between PM hexagonal and FM orthorhombic phase can be obtained in a large CTW by altering the CoNiGe-content. Due to the large ΔM between two phases, the observed MST can be induced by the external magnetic field at room temperature (RT). The effect is accompanied by a large magnetocaloric effect, indicating the potential applications in magnetic cooling refrigerator.

## Results

### Structural characterization

**Figure 1:**
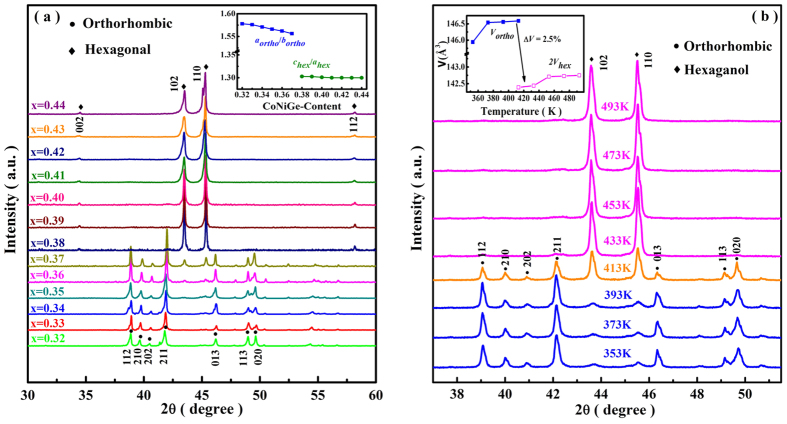
Structural analysis of the phase transitions. (**a**) Composition dependent XRD patterns of (MnNiSi)_1−x_(CoNiGe)_x_ measured at RT. Inset: *a*_*ortho*_/*b*_*ortho*_ and *c*_*hex*_/*a*_*hex*_ ratios. (**b**) Temperature-dependent XRD of (MnNiSi)_0.66_(CoNiGe)_0.34_ from 353 to 493 K. Inset: cell volumes from 353 to 493 K for (MnNiSi)_0.66_(CoNiGe)_0.34_.

Figure 1(a) shows X-ray diffraction (XRD) patterns of (MnNiSi)_1−x_(CoNiGe)_x_ (x = 0.32, 0.33, 0.34, 0.35, 0.36, 0.37, 0.38, 0.39, 0.40, 0.41, 0.42, 0.43 and 0.44) alloys measured at RT. It is reported that the stoichiometric MnNiSi exhibits the orthorhombic TiNiSi structure at RT^24^, while CoNiGe possesses the stable hexagonal Ni_2_In structure^42^. With the increase of CoNiGe-content, the structure of (MnNiSi)_1−x_(CoNiGe)_x_ samples at RT changes from the orthorhombic TiNiSi to hexagonal Ni_2_In phase and no other impurity phase is found. For the samples with x ≤ 0.36, TiNiSi structure dominates at RT; while for x = 0.37, the XRD pattern indicates the coexistence of TiNiSi and Ni_2_In structures; when x ≥ 0.38, the single Ni_2_In structure is observed. XRD results suggest that the introduction of CoNiGe will stabilize Ni_2_In structure and lower down T_t_ from 1200 K to below RT^24^. From the crystallographic point of view, TiNiSi structure can be understood as the distortion of Ni_2_In structure and the axes of the two structures are related as *a*_*ortho*_* = c*_*hex*_, *b*_*ortho*_* = a*_*hex*_ and *c*_*ortho*_ = 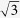
*a*_*hex*_^25^. It is known that the stability of Ni_2_In structure is associated with the low *c*_*hex*_*/a*_*hex*_ (*a*_*ortho*_*/b*_*ortho*_)^39^,^43^. In (MnNiSi)_1−x_(CoNiGe)_x_ system, the value of *a*_*ortho*_*/b*_*ortho*_ (*c*_*hex*_*/a*_*hex*_) decreases with increasing CoNiGe-content, especially at the range of x ≤ 0.36, shown in the inset of Fig. 1(a). It further supports that Co and Ge co-substitution can reduce T_t_.

To further investigate the thermo-induced structural transformation in (MnNiSi)_1−x_(CoNiGe)_x_ system, the temperature-dependent XRD measurement during heating is performed on (MnNiSi)_0.66_(CoNiGe)_0.34_. As shown in Fig. 1(b), (MnNiSi)_0.66_(CoNiGe)_0.34_ exhibits TiNiSi structure at below 390 K, while becomes pure Ni_2_In structure at above 433 K. When the temperature is 413 K, two structures coexist, suggesting that TiNiSi structure transits to Ni_2_In structure with increasing temperature. According to XRD analysis, the temperature-dependent unit-cell volume is calculated, shown in the inset of Fig. 1(b). A large decrease of 2.5% in unit-cell volume is observed during the structural transformation on heating, indicating a remarkable atomic displacement during the structural reconstruction.

### MST and magnetic phase diagram

**Figure 2:**
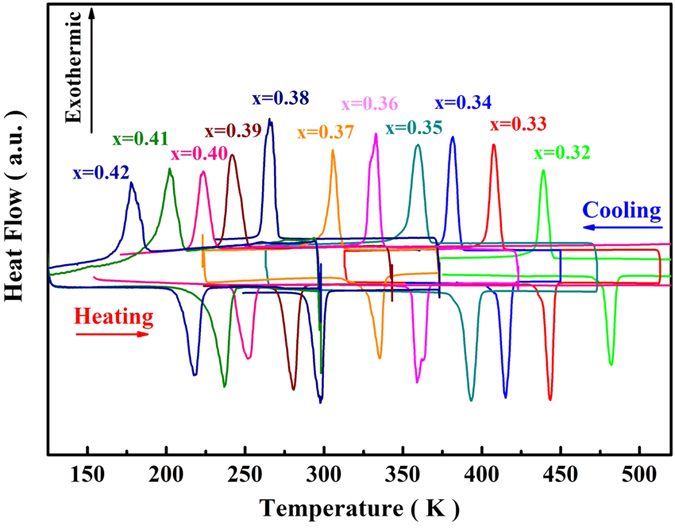
DSC curves for (MnNiSi)_1−x_(CoNiGe)_x_ (x = 0.32, 0.33, 0.34, 0.35, 0.36, 0.37, 0.38, 0.39, 0.40, 0.41 and 0.42) samples. The red and blue arrows denote the heating and cooling processes, respectively.

In order to confirm the CoNiGe-content dependent T_t_, DSC measurements of (MnNiSi)_1−x_(CoNiGe)_x_ (x = 0.32, 0.33, 0.34, 0.35, 0.36, 0.37, 0.38, 0.39, 0.40, 0.41, 0.42) were carried out upon cooling and heating with a ramp rate of 10 K/min, which are shown in Fig. 2. The observed large endothermic/exothermic peaks during heating/cooling cycles are associated with the latent heat of the first-order structural transitions between TiNiSi and Ni_2_In structures. The thermal hysteresis between heating and cooling cycles signifies the first-order nature of structural transformation. For the sample with x = 0.34, the endothermic peak appears at 410 K, which agrees well with the temperature-dependent XRD analysis. It is also found that the endothermic and exothermic peaks shift towards lower temperatures with the increase of CoNiGe-content. This phenomenon verifies that the introduction of CoNiGe can reduce T_t_ of MnNiSi from 1200 K to be below RT.

**Figure 3:**
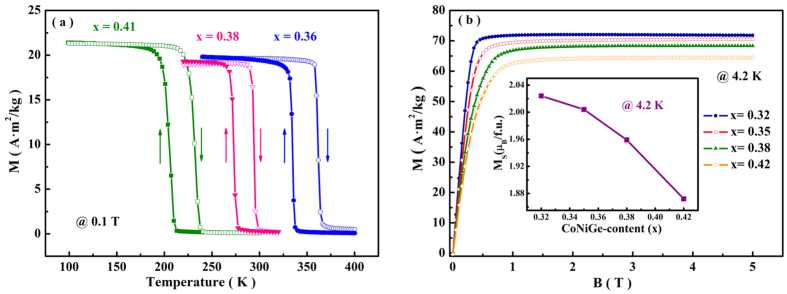
(**a**) M-T curves under the field of 0.1 T during heating and cooling for (MnNiSi)_1−x_(CoNiGe)_x_ (x = 0.36, 0.38 and 0.41). (**b**) Isothermal magnetization curves at T = 4.2 K for (MnNiSi)_1−x_(CoNiGe)_x_ (x = 0.32, 0.35, 0.38 and 0.42). Inset: M_s_ of the samples in the orthorhombic phases.

Since the magnetic properties of MnM′X alloys are sensitive to the Mn-Mn distances^44^, the large distortion of unit-cell during the structure transformation may bring about considerable changes of magnetic states. The temperature dependences of magnetization (M-T) for (MnNiSi)_1−x_(CoNiGe)_x_ (x = 0.36, 0.38 and 0.41) measured during heating and cooling with a ramp rate of 2 K/min at a magnetic field of 0.1 T are shown in Fig. 3(a) (some other representative M-T curves are shown in the supporting information). A sharp magnetic transition from the high-temperature PM to low-temperature FM state is observed during cooling. The transition shifts to the lower temperatures with the increase of CoNiGe-content. The obvious thermal hysteresis, reflecting the irreversibility between cooling and heating cycles, suggests the first-order nature of the transition. T_t_, here defined as the temperature where |dM/dT| is the maximum, agrees well with the DSC measurement. These results prove that the studied samples experience MST between FM orthorhombic and PM hexagonal phase as the temperature changes. While it is worth noting that Mn atoms carry the majority of magnetic moments in MnNiSi alloys^35^. To investigate the saturated magnetization, M_s_, of MnNiSi-CoNiGe system, M-B curves of some samples are measured at 4.2 K in Fig. 3(b). As shown in the inset, M_s_ decreases with increasing CoNiGe-content, and it is lower than that of stoichiometric MnNiSi (2.62 μ_B_/f.u.)^35^. It indicates that the substitution of large-moment Mn atoms by small-moment Co atoms modifies the exchange interactions between Mn-Mn atoms^37^.

**Figure 4:**
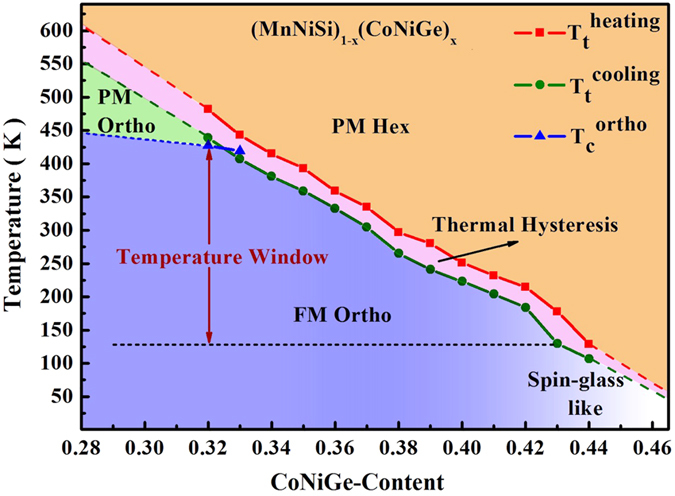
Magnetic and structural phase diagram of (MnNiSi)_1−x_(CoNiGe)_x_ system.

According to DSC and magnetic measurements, the structural and magnetic phase diagram of (MnNiSi)_1−x_(CoNiGe)_x_ system is obtained, as shown in Fig. 4. The sample with x = 0.32 transits from PM hexagonal to PM orthorhombic phase at 450 K, then to FM orthorhombic phase at 425 K during cooling. Upon the further increase of CoNiGe-content to 0.43, T_t_ continuously decreases to 125 K. When x is higher than 0.43, MST disappears and a weak magnetic spin-glass-like state is found, similar as Mn_1−x_Fe_x_NiGe^16^. Thus, a CTW ranged from 425 to 125 K is established in (MnNiSi)_1−x_(CoNiGe)_x_ system, where alloys undergo a tunable MST coupled with a magnetic transition from PM to FM state.

### Magnetic-field-inducing MST and magnetocaloric effect

**Figure 5:**
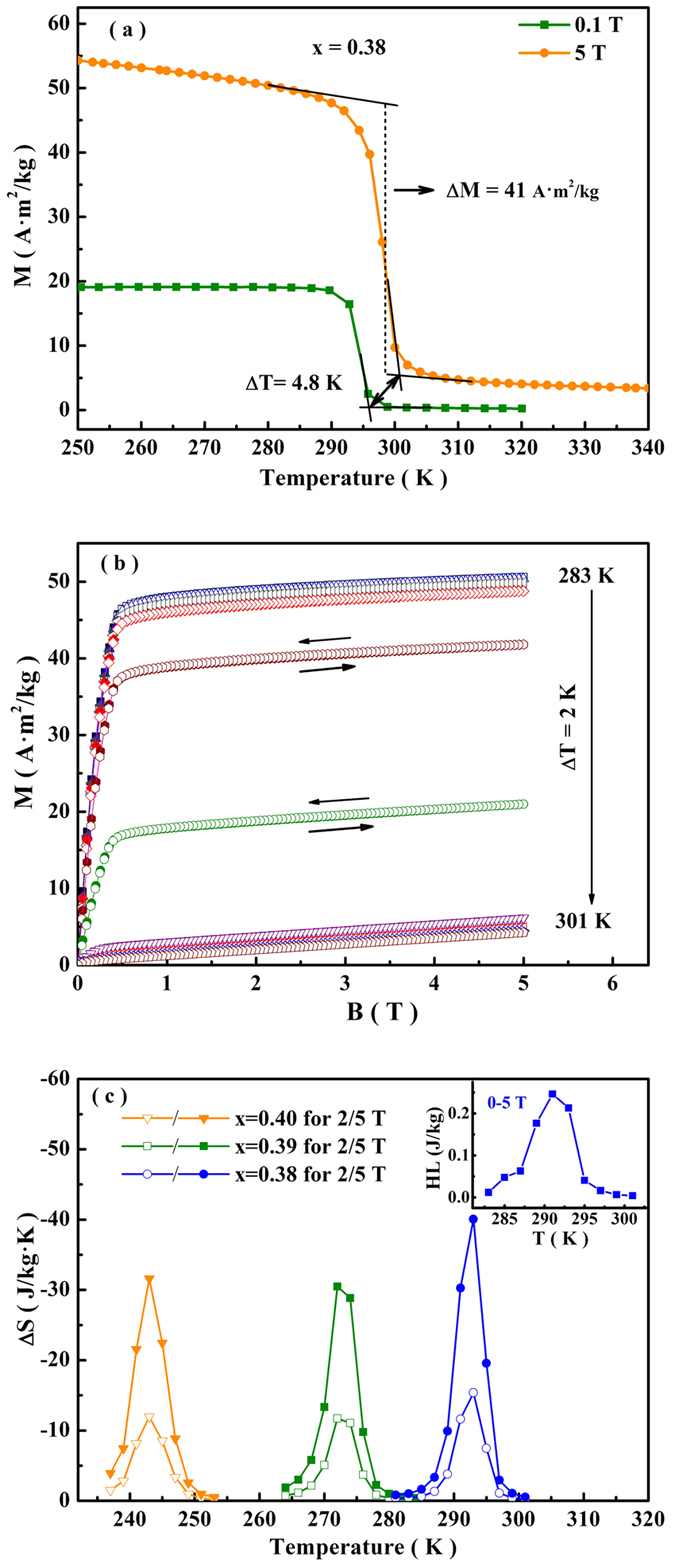
(**a**) M-T curves of (MnNiSi)_0.62_(CoNiGe)_0.38_ for applied fields B = 0.1 T and 5 T. (**b**) Isothermal M-B curves measured around T_t_ for the sample with x = 0.38 (**c**) Temperature dependence of ΔS in the field changes of 0–2 T and 0–5 T for the samples x = 0.38, 0.39 and 0.40. Inset: magnetic hysteresis loss of sample with x = 0.38.

In the case of Ni-Mn based ferromagnetic shape memory alloys, due to the large ΔM between FM austenite and weak magnetic martensite, the austenite is energetically more favorable in the magnetic field, giving rise to the decrease of T_t_ and the magnetic-field-inducing MST from the martensite to austenite^1^. Similarly, in (MnNiSi)_1−x_(CoNiGe)_x_ system with a first-order PM-FM transition, the magnetic field will stabilize FM TiNiSi structure and lead to a MST from the hexagonal to orthorhombic phase. For the sample with x = 0.38, T_t_ increases 4.8 K under a magnetic field of 5 T, indicating that MST can be induced by the magnetic field (Fig. 5(a)). Due to the discontinuities of spin and lattice, MST is associated with a large magnetic entropy change (ΔS). It is known that the maximum value of ΔS can be estimated from M-T curves measured at different constant fields (here, B = 0.1 and 5 T, respectively) using the Clausius-Clapeyron equation:


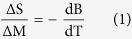


For (MnNiSi)_0.62_(CoNiGe)_0.38_, the calculated maximum value of ΔS is −41.8 J/(kg·K), where ΔM = 41 A·m^2^/kg, ΔB = 4.9 T and ΔT = 4.8 K (Fig. 5(a)). To further confirm the magnetocaloric effect during the transition, ΔS in the heating process is also calculated from the isothermal magnetization curves (M-B curves in Fig. 5(b)) using the Maxwell relation. As shown in Fig. 5(c), the maximum value of ΔS is −40.3 J/(kg·K) at 295 K, which agrees with the value obtained by Clausius-Clapeyron equation. In (MnNiSi)_1−x_(CoNiGe)_x_ system, the large CTW offers the possibility to obtain large ΔS values in the temperature range of nearly 300 K. As an example, the substitution levels of x = 0.40 and 0.39 give rise to ΔS of −31.7 and −30.5 J/(kg·K) for ΔB = 5 T at 245 and 270 K, respectively (Fig. 5(c)). The observed maximum ΔS is larger than some promising magnetocaloric systems, such as some MnM′Ge-based systems^16^,^30^,^33^, Ni-Mn based alloys^7^,^8^ and rare earth-transition metal intermetallic compounds^45^. The effective refrigeration capacity (RC_eff_), which is calculated by subtracting the average hysteresis loss (HL) from the refrigeration capacity (RC) value, is commonly adopted to evaluate magnetocaloric effect^46^. For sample with x = 0.38, RC value is 170.1 J/kg around room temperature, numerically calculated by integrating the area under ΔS-T curves, using the temperatures at half-maximum of the peak as the integration limits. And HL, calculated from the area surrounded by hysteresis loops (M-B curves), is negligible, as shown in the inset of Fig. 5(c). Therefore, RC_eff_ is about 169.8 J/kg for this sample under the field change of 0–5 T. These indicate the potential applications for the RT magnetic cooling refrigerator. Besides, as mentioned above, the MnNiSi-CoNiGe system undergoes large changes of lattice parameters and unit volume during the transition (Fig. 1(a)), which can be induced by the applied magnetic field. This may be utilized for the strain-based applications^16^.

## Discussion

The observed MST between FM orthorhombic and PM hexagonal phase is achieved by adjusting T_t_ into the CTW. However, as mentioned above, T_t_ of MnNiSi is as high as 1200 K and cannot be efficiently decreased by the conventional methods^36^,^37^. Here, the question is why Co and Ge co-substitution can sharply reduce T_t_ to below T_C_, leading to the observed MST. In MnM′X alloys, covalent bonds form between M′ and X atoms and between the neighbouring Mn and Mn atoms both in TiNiSi and Ni_2_In structures^16^,^28^,^29^. These covalent bonds act as skeletons, stabilizing the crystalline structure^16^,^29^,^47^. The structure transformation can be understood as a competition between the strengths of covalent bonds in TiNiSi and Ni_2_In structures^16^,^34^,^47^. Thus, altering the strength of covalent bonds can influence T_t_. For the system with M′ = Ni, T_t_ of MnNiGe lies around 470 K^24^,^25^, which is 730 K lower than that of MnNiSi^24^. This suggests that altering the main-group elements can influence the strength of covalent bond, leading to the change of T_t_. Similar phenomenon is found in Al substituted MnNiGe alloy^48^. Except for altering elements which form M′-X covalent bond, the decrease of T_t_ is also observed when Mn is replaced by other 3*d*-atoms, such as Mn_1−x_Fe_x_CoGe alloy^49^. Based on the site-preference rule^50^, Fe atoms occupy Mn sites and induce lattice change in *c* axis. This change will influence the separation of Mn atoms, leading to the enhancement of the strength of Mn-Mn covalent bonds, which is helpful to stabilizing the Ni_2_In structure. For MnNiSi, T_t_ cannot be efficiently reduced by the single-element substitution^36^,^37^. However, when CoNiGe is introduced, Co atoms occupy 3*d*-atom Mn sites, Ge atoms occupy main-group Si sites, and T_t_ decreases by almost 1000 K with increasing CoNiGe-content.

From the view of applications, a large CTW enables tunable magnetoresponsive effects in a wide temperature range. In this (MnNiSi)_1−x_(CoNiGe)_x_ system, the CTW is as large as 300 K, which is comparable to the previously reported systems, such as MnCoGe-based system^23^, Mn_1−x_Co_x_NiGe^33^, MnNiGe:Fe system^16^, (Mn, Fe)Ni(Ge, Si)^34^ and Gd-Si-Ge alloys^51^. It is known that the realization of PM-FM-type MST should meet the condition that T_t_ can be tuned into the temperature window between the magnetic-ordering temperatures of orthorhombic and hexagonal phases. In (MnNiSi)_1−x_(CoNiGe)_x_ system, T_C_ of orthorhombic phase is around 425 K (shown in Fig. S1). However, with the increase of CoNiGe-content, a spin-glass-like state appears and the magnetic order-disorder transition of hexagonal phase is still not observed (Fig. S1). This phenomenon indicates that the magnetic ordering temperature of hexagonal phase is lower than 125 K. Therefore, a large CTW between 425 and 125 K is established in (MnNiSi)_1−x_(CoNiGe)_x_ system.

## Conclusions

To summarize, we have successfully realized a PM-FM magnetostructural coupling in (MnNiSi)_1−x_(CoNiGe)_x_ system. By introducing CoNiGe which possesses a stable Ni_2_In structure, T_t_ decreases sharply, resulting in the first-order MST between FM orthorhombic and PM hexagonal phase. This is due to the enhancement of covalent bonds which modifies the relative stability of two structures. Besides, a large CTW of 300 K is established, which will benefit the multifunctional applications of the materials over a wide temperature range. Due to the large ΔM during the transition, MST can not only be induced by temperature, but also by magnetic field. Large, tunable magnetocaloric effect generated from MST is obtained. The magnetic entropy change of (MnNiSi)_0.62_(CoNiGe)_0.38_ reaches −40.3 J/(kg·K) under the field change of 0–5 T around RT, suggesting the potential applications in magnetic cooling refrigerator.

## Methods

The samples with nominal compositions of (MnNiSi)_1−x_(CoNiGe)_x_ (x = 0.32, 0.33, 0.34, 0.35, 0.36, 0.37, 0.38, 0.39, 0.40, 0.41, 0.42, 0.43 and 0.44) were prepared by arc-melting the appropriate amounts of raw materials in a water-cooled copper crucible under a high purity argon atmosphere for three times. As-cast ingots were annealed in vacuumed quartz tubes at 1073 K for four days before quenched into the cold water.

The crystal structures of the specimens were identified by X-ray diffraction (XRD, Bruker, D8 Advance) analysis with Cu-Kα radiation. The structural transitions were investigated by differential scanning calorimetry (DSC, Mettler Toledo, DSC 3).

Magnetic measurements were performed with vibrating sample magnetometer (VSM, LakeShore, 7407) and Physical Property Measurement System (PPMS, Quantum Design, Dynacool). Isothermal magnetic entropy change (ΔS) was calculated from the isothermal magnetization curves using Maxwell equation (2):


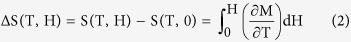


To avoid the irreversibility in the magnetic-field-induced first-order MST, isothermal magnetization curves were measured using a so-called loop process^52^: in the heating process, the isothermal magnetization were measured with a temperature interval of 2 K in the magnetic field variation from 0 to 5 T. For each M-B curve, the samples were initially cooled down to complete orthorhombic region (for our samples, it’s around 150 K) at 5 K/min. Then the samples were heated slowly to the measuring temperature at 3 K/min. To guarantee the temperature stability of measurement, a waiting time of 300 s was hold at the initial and targeted temperatures. Besides, for the samples with x = 0.38, 0.39 and 0.40, the highest loop temperatures were 301, 288 and 261 K, respectively.

## Additional Information

**How to cite this article**: Liu, J. *et al.* Realization of magnetostructural coupling by modifying structural transitions in MnNiSi-CoNiGe system with a wide Curie-temperature window. *Sci. Rep.* **6**, 23386; doi: 10.1038/srep23386 (2016).

## Supplementary Material

Supplementary Information

## References

[b1] Kainuma R. *et al.* Magnetic-field-induced shape recovery by reverse phase transformation. Nature 439, 957–960 (2006). 16495995 10.1038/nature04493

[b2] Ullakko K., Huang J. K., Kantner C., O’Handley R. C. & Kokorin V. V. Large magnetic-field-induced strains in Ni_2_MnGa single crystal. Appl. Phys. Lett. 69, 1966–1968 (1996).

[b3] Chmielus M., Zhang X. X., Witherspoon C., Dunand D. C. & Mullner P. Giant magnetic-field-induced strains in polycrystalline Ni-Mn-Ga foams. Nat. Mater. 8, 863–866 (2009). 19749769 10.1038/nmat2527

[b4] Sharma V. K., Chattopadhyay M. K., Shaeb K. H. B., Chouhan A. S. & Roy B. Large magnetoresistance in Ni_50_Mn_34_In_16_ alloy. Appl. Phys. Lett. 89, 222509 (2006).

[b5] Ma S. C. *et al.* Peculiarity of magnetoresistance in high pressure annealed Ni_43_Mn_41_Co_5_Sn_11_ alloy. Appl. Phys. Lett. 102, 032407 (2013).

[b6] Pecharsky V. K. & Gschneidner K. A. Jr. Giant magnetocaloric effect in Gd_5_(Si_2_Ge_2_). Phys. Rev. Lett. 78, 4494 (1997). 10.1103/PhysRevLett.84.4617 10990754

[b7] Krenke T. *et al.* Inverse magnetocaloric effect in ferromagnetic Ni-Mn-Sn alloys. Nat. Mater. 4, 450–454 (2005). 15895096 10.1038/nmat1395

[b8] Liu J., Gottschall T., Skokov K. P., Moore J. D. & Gutfleisch O. Giant magnetocaloric effect driven by structural transitions. Nat. Mater. 11, 620–626 (2012). 22635044 10.1038/nmat3334

[b9] Zhang Y. *et al.* Enhanced magnetic refrigeration properties in Mn-rich Ni-Mn-Sn ribbons by optimal annealing. Sci. Rep. 5, 11010 (2015). 26055884 10.1038/srep11010 PMC4460723

[b10] Sarawate N. & Dapino M. Experimental characterization of the sensor effect in ferromagnetic shape memory Ni-Mn-Ga. Appl. Phys. Lett. 88, 121923 (2006).

[b11] Karaca H. E. *et al.* Magnetic field-induced phase transformation in NiMnCoIn magnetic shape-memory alloys—a new actuation mechanism with large work output. Adv. Funct. Mater. 19, 983–998 (2009).

[b12] Liu J., Scheerbaum N., Weiss S. K. & Gutfleisch O. NiMn-Based alloys and composites for magnetically controlled dampers and actuators. Adv. Eng. Mater. 14, 653–667 (2012).

[b13] Karaman I., Basaran B., Karaca H. E., Karsilayan A. I. & Chumlyakov Y. I. Energy harvesting using martensite variant reorientation mechanism in a NiMnGa magnetic shape memory alloy. Appl. Phys. Lett. 90, 172505 (2007).

[b14] Gschneidner K. A. Jr., Pecharsky V. K. & Tsokol A. O. Recent developments in magnetocaloric materials. Rep. Progr. Phys. 68, 1479–1539 (2005).

[b15] Gutfleisch O. *et al.* Magnetic materials and devices for the 21st century: stronger, lighter, and more energy efficient. Adv. Mater. 23, 821–842 (2011). 21294168 10.1002/adma.201002180

[b16] Liu E. K. *et al.* Stable magnetostructural coupling with tunable magnetoresponsive effects in hexagonal ferromagnets. Nat. Commun. 3, 873 (2012). 22643900 10.1038/ncomms1868

[b17] Koyama K., Sakai M., Kanomata T. & Watanabe K. Field-induced martensitic transformation in new ferromagnetic shape memory compound Mn_1.07_Co_0.92_Ge. Jpn. J. Appl. Phys. 43, 8036–8039 (2004).

[b18] Krenke T. *et al.* Ferromagnetism in the austenitic and martensitic states of Ni-Mn-In alloys. Phys. Rev. B 73, 174413 (2006).

[b19] Mañosa L. *et al.* Giant solid-state barocaloric effect in the Ni-Mn-In magnetic shape-memory alloy. Nat. Mater. 9, 478–481 (2010). 20364140 10.1038/nmat2731

[b20] Zhang C. L. *et al.* Magnetostructural phase transition and magnetocaloric effect in off-stoichiometric Mn_1.9-x_Ni_x_Ge alloys. Appl. Phys. Lett. 93, 122505 (2008).

[b21] Trung N. T. *et al.* From single- to double-first-order magnetic phase transition in magnetocaloric Mn_1−x_Cr_x_CoGe compounds. Appl. Phys. Lett. 96, 162507 (2010).

[b22] Trung N. T., Zhang L., Caron L., Buschow K. H. J. & Brück E. Giant magnetocaloric effects by tailoring the phase transitions. Appl. Phys. Lett. 96, 172504 (2010).

[b23] Liu E. K. *et al.* Vacancy-tuned paramagnetic/ferromagnetic martensitic transformation in Mn-poor Mn_1−x_CoGe alloys. Europhys. Lett. 91, 17003 (2010).

[b24] Johnson V. Diffusionless orthorhombic to hexagonal transitions in ternary silicides and germanides. Inorg. Chem. 14, 1117–1120 (1975).

[b25] Bażela W., Szytuła A., Todorovć J., Tomkowicz Z. & Zięba A. Crystal and magnetic structure of NiMnGe. Phys. Status Solidi A 38, 721–729 (1976).

[b26] Fjellvåg H. & Andresen A. F. On the crystal structure and magnetic properties of MnNiGe. J. Magn. Magn. Mater. 50, 291–297 (1985).

[b27] Kanomata T. *et al.* Magneto-volume effect of MnCo_1−x_Ge (00.2). J. Magn. Magn. Mater. 131, 140–144 (1995).

[b28] Kanematsu K. Covalent bond and spin scheme in the intermetallic compound with B8_2_ type. Jpn. J. Appl. Phys. 17, 85–93 (1962).

[b29] Austin A. E. & Adelson E. X-ray spectroscopic studies of bonding in transition metal germanides. J. Solid State Chem. 1, 229–236 (1970).

[b30] Choudhury D., Suzuki T., Tokura Y. & Taguchi Y. Tuning structural instability toward enhanced magnetocaloric effect around room temperature in MnCo_1−x_Zn_x_Ge. Sci. Rep. 4, 7544 (2014). 25519919 10.1038/srep07544 PMC4269893

[b31] Samanta T., Dubenko I., Quetz A., Stadler S. & Ali N. Giant magnetocaloric effects near room temperature in Mn_1−x_Cu_x_CoGe. Appl. Phys. Lett. 101, 242405 (2012).

[b32] Caron L., Trung N. T. & Brück E. Pressure-tuned magnetocaloric effect in Mn_0.93_Cr_0.07_CoGe. Phys. Rev. B 84, 020414(R) (2011).

[b33] Liu E. K. *et al.* Giant magnetocaloric effect in isostructural MnNiGe-CoNiGe system by establishing a Curie-temperature window. Appl. Phys. Lett. 102, 122405 (2013).

[b34] Wei Z. Y. *et al.* Unprecedentedly wide Curie-temperature windows as phase-transition design platform for tunable magneto-multifunctional materials. Adv. Electron. Mater. 1, 1500076 (2015).

[b35] Johnson V. & Fredbrick C. G. Magnetic and crystallographic properties of ternary manganese silicides with ordered Co_2_P structure. Phys. Status Solidi A 20, 331 (1973).

[b36] Duraj R. & Zach R. Magnetic phase transitions in NiMnSi_n_Ge_1-n_ and NiMn_1-t_Ti_t_Ge systems under pressure. J. Magn. Magn. Mater. 73, 69–78 (1988).

[b37] Li Y. *et al.* Structural transitions, magnetic properties, and electronic structures of Co(Fe)-doped MnNiSi compounds. J. Appl. Phys. 117, 17C117 (2015).

[b38] Zhang C. L. *et al.* The tunable magnetostructural transition in MnNiSi-FeNiGe system. Appl. Phys. Lett. 103, 132411 (2013).

[b39] Samanta T. *et al.* Hydrostatic pressure-induced modifications of structural transitions lead to large enhancements of magnetocaloric effects in MnNiSi-based systems. Phys. Rev. B 91, 020401(R) (2015).

[b40] Samanta T. *et al.* Effects of hydrostatic pressure on magnetostructural transitions and magnetocaloric properties in (MnNiSi)_1−x_(FeCoGe)_x_. J. Appl. Phys. 117, 123911 (2015).

[b41] Zhang C. L. *et al.* Magnetostructural transition and magnetocaloric effect in MnNiSi-Fe_2_Ge system. Appl. Phys. Lett. 107, 212403 (2015).

[b42] Kanematsu K., Ohoyama T. & Yasukochi K. Magnetic property of (Co, Ni)_1.67_Ge. J. Phys. Soc. Jpn. 17, 932 (1962).

[b43] Samanta T. *et al.* Mn_1−x_Fe_x_CoGe: A strongly correlated metal in the proximity of a noncollinear ferromagnetic state. Appl. Phys. Lett. 103, 042408 (2013).

[b44] Barcza A., Gercsi Z., Knight K. S. & Sandeman K. G. Giant magnetoelastic coupling in a metallic helical metamagnet. Phys. Rev. Lett. 104, 247202 (2010). 20867331 10.1103/PhysRevLett.104.247202

[b45] Zhang H. *et al.* Giant rotating magnetocaloric effect induced by highly texturing in polycrystalline DyNiSi compound. Sci. Rep. 5, 11929 (2015). 26159558 10.1038/srep11929 PMC5155619

[b46] Huang L., Cong D. Y., Suo H. L. & Wang Y. D. Giant magnetic refrigeration capacity near room temperature in Ni_40_Co_10_Mn_40_Sn_10_ multifunctional alloy. Appl. Phys. Lett. 104, 132407 (2014).

[b47] Wei Z. Y. *et al.* Realization of multifunctional shape-memory ferromagnets in all-*d*-metal Heusler phases. Appl. Phys. Lett. 107, 022406 (2015).

[b48] Samanta T. *et al.* Magnetostructural phase transitions and magnetocaloric effects in MnNiGe_1−x_Al_x_. Appl. Phys. Lett. 100, 052404 (2012).

[b49] Wang Z. L. *et al.* First-order magnetostructural transformation in Fe doped Mn-Co-Ge alloys. J. Alloys Comp. 577, 486–490 (2013).

[b50] Szytuła A. *et al.* Crystal and magnetic structure of CoMnGe, CoFeGe, FeMnGe and NiFeGe. J. Magn. Magn. Mater. 25, 176–186 (1981).

[b51] Pecharsky V. K. & Gschneidner K. A. Jr. Tunable magnetic regenerator alloys with a giant magnetocaloric effect for magnetic refrigeration from ~20 to ~290 K. Appl. Phys. Lett. 70, 3299 (1997).

[b52] Caron L. *et al.* On the determination of the magnetic entropy change in materials with first-order transitions. J. Magn. Magn. Mater. 321, 3559–3566 (2009).

