# Supplementary material for: Realization of magnetostructural coupling by modifying structural transitions in MnNiSi-CoNiGe system with a wide Curie-temperature window
Source: Sci Rep. 2016 Mar 16;6:23386. doi: 10.1038/srep23386 (PMC4793218; doi:10.1038/srep23386)
Supplement: Supplementary Information [file srep23386-s1.pdf]

Realization of magnetostructural coupling by modifying structural transitions in MnNiSi-CoNiGe system with a wide Curie-temperature window

Jun Liu[1], Yuanyuan Gong[1], Guizhou Xu[1], Guo Peng[2], Ishfaq Ahmad Shah[1], Najam ul Hassan[1], & Feng Xu[1,*]

[1]Jiangsu Key Laboratory of Advanced Micro&Nano Materials and Technology, School of Materials Science and Engineering, Nanjing University of Science and Technology, Nanjing 210094, China
[2]Herbert Gleiter Institute of Nanoscience, Nanjing University of Science and Technology, Nanjing 210094, China

*Corresponding author. E-mail: xufeng@njust.edu.cn, Tel: +86-25-84303411.

Supplementary Information

For x = 0.33, the magnetization shows a sharp increase at 405 K in the cooling process, which is corresponding to the thermal induced magnetostructural transformation from the paramagnetic hexagonal to ferromagnetic orthorhombic phase. T_t (405 K) agrees well with the DSC data in the cooling process. However, in the heating process, the magnetization begins to gradually decrease at 408 K, then sharply reduce to 0 when the temperature reaches 440 K. This phenomenon suggests that the sample first undergoes a magnetic ordering-disordering transition of the orthorhombic phase and then experiences a martensitic transformation from the orthorhombic to hexagonal phase. Similar phenomenon is also observed in the sample with x = 0.32. In the heating process, the sample experiences a magnetic ordering-disordering transition followed by a sharp structural transformation from paramagnetic orthorhombic to paramagnetic hexagonal phase, which is shown in Fig. S1(a). Based on the M-T curves for x = 0.32 and 0.33, T_C of orthorhombic phase is confirmed to be 417 and 425 K, respectively. (T_C is defined as the maximum value of dM/dT in the heating process.)

For sample with x = 0.44, it shows the irreversible property and weak-magnetic spin-glass-state, in the case as $Mn_{1-x}NiFe_xGe$[16]. According to the results of DSC and M-T curves, structural and magnetic coupling is established in other samples. And a CTW, as large as 300 K, is obtained.

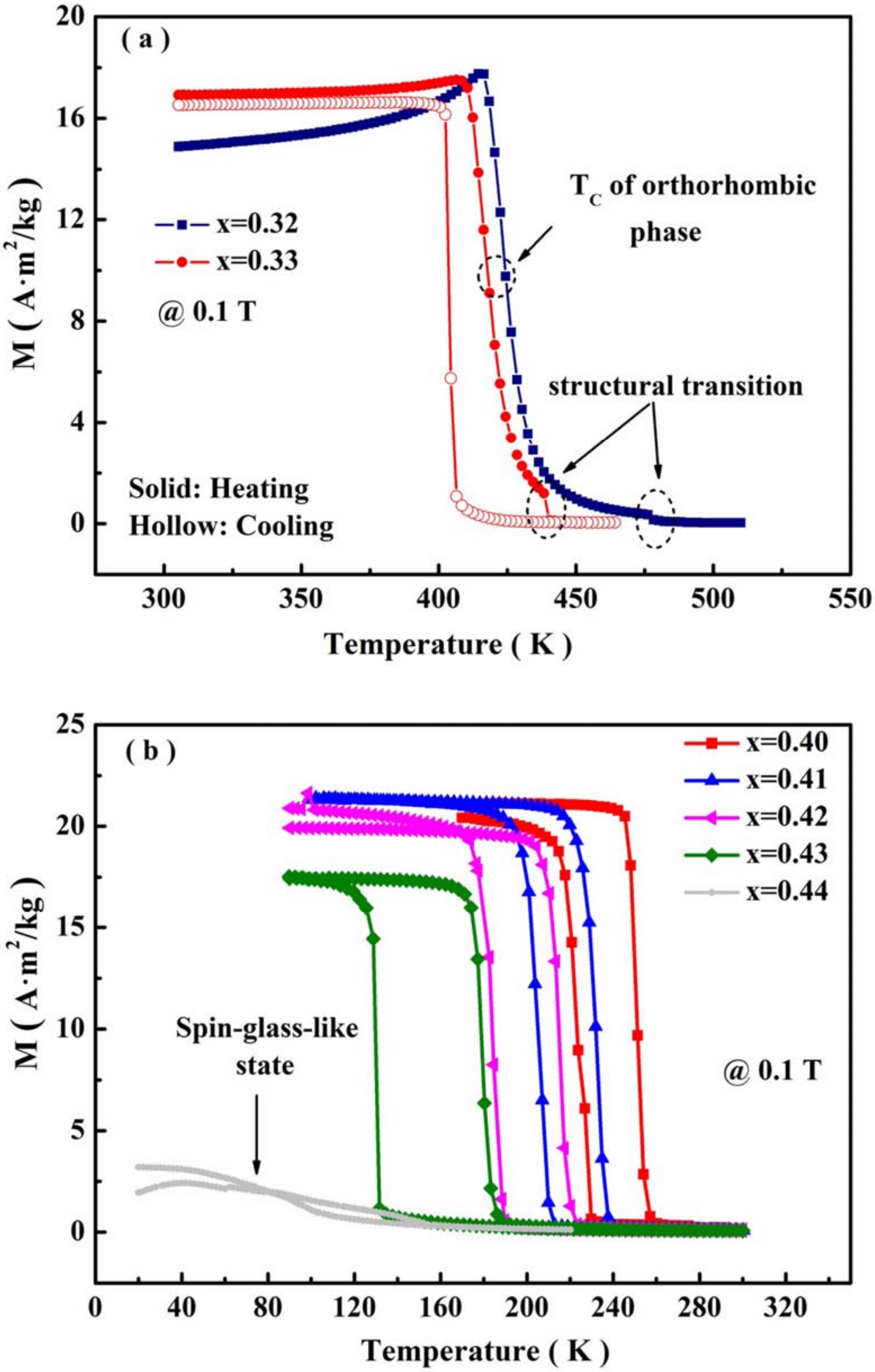

Fig. S1. The thermal magnetic curves (M-T) for $(MnNiSi)_{1-x}(CoNiGe)_x$. (a) M-T curves of samples with x = 0.32 and 0.33. (b) M-T curves of samples from x = 0.40 to 0.44.
